# Right ventricular outflow tract deployment of stents in the management of tetralogy with hypoplastic pulmonary arteries

**DOI:** 10.1186/1749-8090-8-S1-O312

**Published:** 2013-09-11

**Authors:** A Nohkrin, K Drozdovski, A Bashkevitch, A Savchuk, E Karalkova, I Turchinova, E Karatshan, W Novick

**Affiliations:** 1 Pediatric Cardiac Surgery, Research Institute for Complex Issues of Cardiovascular Diseases, Siberian Branch of the Russian Academy of Medical Sciences, Kemerovo, Russia; 2 Pediatric Cardiac Surgery, National Children's Cardiac Surgical Center, Minsk, Belarus; 3 Pediatric Cardiology, National Children's Cardiac Surgical Center, Minsk, Belarus; 4 Pediatric Cardiology, Research Institute for Complex Issues of Cardiovascular Diseases, Siberian Branch of the Russian Academy of Medical Sciences, Kemerovo, Russia; 5 Department of Surgery, University of Tennessee Health Sciences Center and International Children's Heart Foundation, Memphis, TN, USA

## Objective

Patients with tetralogy or double outlet right ventricle with pulmonary stenosis who present with cyanosis before 4 months of age remain a treatment challenge. Those patients presenting as newborns are especially difficult to manage. We have introduced a coronary stent into the right ventricular outflow tract (RVOT) as an alternative to performing a systemic to pulmonary artery shunt.

## Methods

The databases in Minsk and Kemerovo were queried to identify all patients undergoing stent deployment in the RVOT. Additionally all patients less than 4 months of age receiving a systemic to pulmonary shunt for TOF or DORV/PS were identified. A total of 35 patients were identified. The data for those receiving stents was compared to those receiving shunts.

## Results

Twelve (12) children received a stent and 23 received shunts. Age (days) and weight (kg) respectively were 59.2 ± 30.9, 3.7 ± 0.9 for stent patients and 64.2 ± 40.9, 3.9 ± 1.3, for shunt patients (p, NS for age and weight). Pre-procedural saturations and pulmonary artery size (mean branch size in mm) were 74.6% ± 13.6, 4.8 ± 1.3 for shunts and 76.7 ± 11.2, 3.9 ± 0.8 for stents (p, NS sats, p = 0.017, size). Secondary procedures were required in 3/12 stent patients and 6/23 shunt patients. Initial procedure mortality was 0/12 (0%) stents and 2/23 (8.7%) shunts. The time interval between initial procedure and complete repair was 17.7 ± 5.2 months for shunt patients and 6.5 ± 4.2 months for stent patients (p < 0.001). Repair type was trans-annular patch or right ventricular pulmonary artery conduit in 89% (16/18) shunt patients and all patients with stents (9/9) had a trans-annular patch. Post repair procedures consisted of coiling collaterals in 2/9 with stents and none with shunts. Survival through complete repair was 100% (9/9) in stents and 85% (17/20) in the shunts.

## Conclusion

Stents placed in the RVOT of infants with tetralogy can be performed with low mortality and improve total survival.

